# Interleukin-1 receptor antagonist inhibits matastatic potential by down-regulating CXCL12/CXCR4 signaling axis in colorectal cancer

**DOI:** 10.1186/s12964-021-00804-0

**Published:** 2021-12-20

**Authors:** Jiachi Ma, Wanqing Liang, Yaosheng Qiang, Lei Li, Jun Du, Chengwu Pan, Bangling Chen, Chensong Zhang, Yuzhong Chen, Qingkang Wang

**Affiliations:** 1 Department of Oncological Surgery, The First Affiliated Hospital of Bengbu Medical College, Bengbu Medical College, No. 287 Changhuai Road, Longzihu District, Bengbu, 233000 Anhui China; 2 Department of General Surgery, The First Hospital of Lanzhou University, Lanzhou University, No. 1 Donggangxi Road, Chengguan District, Lanzhou, 730000 Gansu China

**Keywords:** Interleukin-1 receptor antagonist, CXCR4-CXCL12 axis, Colorectal cancer, Metastasis

## Abstract

**Background:**

The aim of this study was to investigate the co-operative role of CXCR4/CXCL12 axis and IL-1Ra in metastatic processes mechanism by interactions between colorectal cancer cells and stromal cells in their microenvironment.

**Methods:**

Expression of IL-1α, interleukin-1 receptor type I (IL-1 RI), CXCL12 and CXCR4 mRNA and proteins were determined by RT-PCR and Western blot. The effect of secreted level of CXCL12 by IL-1Ra on fibroblasts was measured by ELISA. CXCL12 regulate metastatic potential of colorectal cancer was evaluated by proliferation, invasion and angiogenesis assays, respectively, in which invasion and angiogenesis assays used an in vitro system consisting of co-cultured colorectal cells and stromal cells.

**Results:**

IL-1α was expressed in high liver metastatic colorectal cancer cell lines (HT-29 and WiDr). The colorectal cancer cell-derived IL-1α and rIL-1α significantly promoted CXCL12 expression by fibroblasts, and this enhancing effect can be significantly inhibited by IL-1Ra (*P* < 0.01)*.* CXCL12 not only enhanced the migration and proliferation of human umbilical vein endothelial cells, but also significantly enhanced angiogenesis (*P* < 0.01). Furthermore, the high liver-metastatic colorectal cancer cell line (HT-29), which secretes IL-1α, significantly enhanced angiogenesis compared to the low liver-metastatic cell line (CaCo-2), which does not produce IL-1α (*P* < 0.01). On the contrary, IL-1Ra can significantly inhibit migration, proliferation and angiogenesis (*P* < 0.01).

**Conclusion:**

Autocrine IL-1α and paracrine CXCL12 co-enhances the metastatic potential of colorectal cancer cells; IL-1Ra can inhibit the metastatic potential of colorectal cancer cells via decrease IL-1α/CXCR4/CXCL12 signaling pathways.

**Graphical Abstract:**

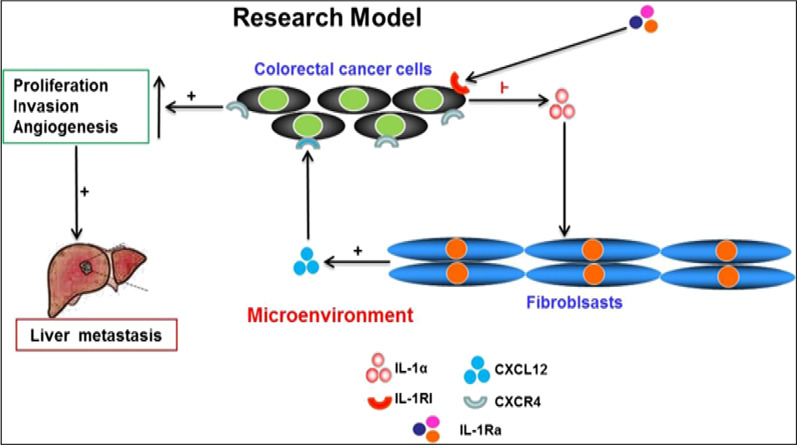

Video Abstract

**Supplementary Information:**

The online version contains supplementary material available at 10.1186/s12964-021-00804-0.

## Background

Colorectal cancer (colorectal carcinoma, CRC) is one of the most common malignant tumors in the world, with the third highest incidence of male cancers and the second highest female cancer rate. Its fatality rate ranks second among cancer-related deaths, with more than 940,000 deaths per year. Globally, colorectal cancer has an incidence rate of 24.26/100,000, and the number of new cases each year is about 1.93 million, accounting for 10.2% of all new cases of malignant tumors, ranking third [1]. Hematogenous metastasis is one of the most common forms of distant metastasis of colorectal cancer, and the liver is one of their main targets, which is also the main cause of death in patients with colorectal cancer. In addition, more than 1/3 of patients die within five years after the initial diagnosis, the primary cause of which also being liver metastasis [2]. Liver metastasis is one of the key points and difficulties in the treatment of colorectal cancer, as well as the key factors affecting the prognosis for patients.

CXCL12, a member of the chemokine family, also known as stromal cell-derived factor-1, is a multipotent chemokine, which is widely expressed in brain, lung, colon, heart and liver. It can stimulate a variety of signal transduction and has chemotactic effect on tumor cells which can express its corresponding receptor CXCR4, making tumors form specific metastatic foci [3]. Studies have shown that CXCL12 is expressed in the cytoplasm and cell membrane of colon cancer cells, as well as in fibroblasts in tumor stroma. Its expression in colorectal cancer cells and disease site is one of the important factors affecting prognosis. Its high expression in colon cancer is related to advanced disease and low survival rate of patients [4]. Our previous studies have found that CXCL12 can also enhance the invasiveness of colon cancer cells and significantly promote the proliferation and migration of human umbilical vein endothelial cells [5]. It has been shown that metastasis occurs through angiogenesis, since newly formed blood vessels are able to carry oxygen and nutrients to the tumor and also stimulate the growth of cancer cells [6]. C-X-C chemokine receptor 4 (CXCR4), the main receptor of CXCL12, is a G protein coupled receptor, just like CXCL12, which is also widely expressed in different tissues. It is generally considered to be a membrane receptor protein that retains the signal after localization to the nucleus [7]. At present, it has been found that CXCL12/CXCR4 axis can play an important role in the occurrence, development and metastasis of many kinds of human tumors, including breast cancer, gastric cancer, colorectal cancer, prostate cancer, renal cell carcinoma, ovarian cancer and so on [8, 9], especially in the biological behaviors such as proliferation, adhesion, migration, invasion and metastasis of tumor cells [10]. More and more evidence showed that the activation of CXCL12/CXCR4 axis is related to liver metastasis and poor prognosis of patients with colorectal cancer [11]. It has been proved that after colorectal cancer cells activate the CXCL12/CXCR4 axis, the miRNAs secreted by colorectal cancer cells can be absorbed by macrophages and transform macrophages to M2 phenotype by targeting PTEN. Then M2 polarized macrophages can promote angiogenesis and liver metastasis of colorectal cancer by secreting VEGF [12]. Some in vitro studies showed that CXCL12/CXCR4 promoted the migration and liver metastasis of CRC by upregulating αvβ6 through ERK1/2/Ets-1 [13]. And the overexpression of CXCR4 could increase both AOM/DSS-induced CAC and Apc mutation-driven tumorigenesis and progression [14]. Therefore, the expression of CXCR4 gene in colorectal cancer is also significantly correlated with tumor recurrence, liver metastasis and prognosis of patients [15].

Interleukin-1α (IL-1α) has long been known for its multiple effects on inflammation. The role of inflammation in various stages of cancer development is complex and even opposite, as well as being a major component of the tumor microenvironment [16]. IL-1 can enhance the invasiveness of malignant tumor cells and eventually lead to metastasis by stimulating growth factors, angiogenesis and the movement of tumor cells. However, in some cases, IL-1α can also enhance the immunogenicity of malignant tumor cells, thus reducing the invasiveness of tumors [17]. IL-1α is regulated by members of the family of IL-1 Receptor Type I (IL-1 RI). The regulatory factors that inhibit IL-1α include bait receptor (IL-1R2), receptor antagonist (IL-1Ra), IL-1R8 and anti-inflammatory IL-37. IL-1 plays different roles in the occurrence and development of cancers. It can further induce the secretion of growth factors. These growth factors induces proliferation, promote angiogenesis, macrophage recruitment, invasion and metastasis, so as to accelerate the progression of tumor [18]. TNF-α and IL-1α in tumor microenvironment can produce DNA damage molecules such as ROS and nitric oxide, which induce mutations in colonic epithelium and promote the development of cancer [19, 20]. It has been suggested that IL-1α derived from intestinal epithelial cells plays a leading role in the pathogenesis of superior mesenteric colitis. IL-1α released by damaged endothelial cells may activate monocytes and infiltrating cells in the colon to release inflammatory mediators, which amplifies the initial inflammatory response and promotes the occurrence and metastasis of colorectal cance [21].

The effects of IL-1α and its receptor antagonist IL-1Ra on the migration of colon cancer cells and promoting angiogenesis and the chemotaxis of CXCL12 all play an important role in tumorigenesis and metastasis. However, the molecular mechanism of the synergistic regulation of CXCR4/CXCL12 axis and IL-1Ra on colon cancer metastasis is still not clear. The purpose of this study is to explore the potential of CXCL12 and IL-1Ra to promote the metastasis of colon cancer and its mechanism, to better understand the interaction between colon cancer cells and stromal cells in tumor microenvironment, and to provide new ideas for the treatment of colorectal cancer and inhibition of liver metastasis of colon cancer.

## Methods

### Cell lines and conditions

Four cell lines derived from human colorectal carcinoma were examined: HT-29, WiDr, CaCo-2 and Colo320. All cell lines were obtained from the American Type Culture Collection (Rockville, MD, USA). The HT-29 was cultured in McCoy’s supplemented with 10% fetal bovine serum (FBS). WiDr and CaCo-2 were maintained in minimum essential medium eagle (Sigma Chemical Co., St. Louis, MO, USA) with high glucose and 10% FBS. Colo320 was maintained in RPMI-1640 medium (Sigma Chemical Co.) supplemented with 10% FBS. Fibroblasts were obtained from Lonza Walkersville Inc. (Walkersville, MD) and maintained in FBM-2 medium supplemented with 2% FBS, 1 ng/ml bFGF, and 1 mg/ml insulin. Human umbilical vein endothelial cell (HUVEC) was obtained from Kurabo Co. (Osaka, Japan). HUVEC were culture in HuMedia-EB2 medium supplemented with 2% FBS, 5 ng/ml of basic fibroblast growth factor, 10 µg/ml heparin, 10 ng/ml epidermal growth factor, and 1 µg/ml hydrocortisone according to the supplier’s instructions (Kurabo Co.). Fibroblast was obtained from Lonza (Walkersville, MD) and maintained in FBM-2 medium supplemented with 2% FBS, 1 ng/mL basic fibroblast growth factor, and 1 µg/ml insulin according to the supplier’s instructions. All cells were incubated at 37 °C in a humidified atmosphere of 5% CO_2_ in air.

### Regents and antibodies

Recombinant human CXCL12 and anti-CXCL12 antibody were purchased from R&D systems (Minneapolis, MN), recombinant human IL-1α was provided by Diaclone (Beasancon, France), while recombinant human IL-1 Receptor Antagonist (IL-1Ra) was provided from Pepro Tech EC Ltd (London, UK).

### RT-PCR analysis

Total RNA was extracted from four colorectal cancer cell lines using Isogen Kit (Nippon Gene Tokyo, Japan), and then quantities were determined spectrophotometrieally. Total RNA aliquots (5 µg) were pretreated with Random Hexamers and dNTP Mix were incubated at 65 °C for 5 min, chilled on ice, and then reverse-transcribed into cDNA using the SuperScript III RT System (Invitrogen, San Diego, CA). One µL of cDNA aliquots was used as the templates for PCR. The pairs of forward and reverse primer sets were designed using Primer 3 software. The primer sequences and PCR conditions were described in Table 1. Amplification reactions were performed by a DNA Thermal Cycle (model TP300; Takara PCR Thermal Cycle MP). The amplified DNA fragments were displayed by electrophoresis on 1.5% agarose gels containing ethidium bromide.

**Table 1:** Primer sequence and PCR condition

Gene name	Primer sequences	Tm (°C)	Cycles	Length (bp)	Accession number
IL-1α	F: 5′-AATGACGCCCTCAATCAAAG-3′	54	35	206	NM-000575
	R: 5′-TGGGTATCTCAGGCATCTCC-3′				
IL-1 RI	F: 5′-CGGCAGGAATGTGACAATCG-3′	56	35	178	AH008153.2
	R: 5′-TCTCACCCCTACCTAGTCCC-3′				
CXCL12	F: 5′-TTCCATTTGCAAGGGAAAAG-3′	56	35	236	NM-000609
	R: 5′-ACACACAGCCAGTCAACGAG-3′				
CXCR4	F: 5′-GAAGCTGTTGGCTGAAAAGG-3′	56	35	345	NM-003467
	R: 5′-GAGTCGATGCTGATCCCAAT-3′				

### Western blot analysis

The cells were lysed in lysis buffer [25 mM Tris (pH 7.8) with H_3_PO, 2 mM CDTA, 10 mM DTT, 10% glycerol, 1% Triton® X-100, 2 mM PMSF, 1 mM sodium orthovanadate, and 10 µM leupeptin]. The protein concentrations were measured with a BCA protein assay kit (Pierce, Rockford, USA). The amounts of samples were 30 µg per each lane. The lysates were separated by 10% SDS–polyacrylamide gel electrophoresis, transferred to polyvinylidene membrane (Immobilo PVDF; Nihon Millipore Ltd, Tokyo, Japan). The membrane was incubated in the blocking buffer for 60 min at room temperature. The blocking buffer was consisted of 5% nonfat dry milk dissolved into Tris buffered saline containing 0.1% Tween 20 (TBS-T). After washing the membrane with TBS-T, the membrane was immunoblotted with each primary antibody diluted into 1:1000–2000 overnight at 4 °C. Afterward, membranes were washed with TBS-T three times, and subjected to HRP-conjugated secondary antibody for 60 min at RT temperature. Protein antibody complexes were visualized with an ECL Western blotting detection and analysis system (Amersham Biosciences, Buckinghamshire, UK).

### Design and synthesis of CXCR4 siRNA and its transfection into colorectal cancer cells

The two specific siRNAs were designed according to the gene region gene sequence of human CXCR4 gene, and CXCR4 SiRNA sequences were as follows: 5′-GCCAAG GAGUGCUAAAGAA-3′ and 5′-CCAACACAGAAAUGU-3′; The Control siRNA sequences were 5′-GUAGCAGGGCA UGUAUUUATT-3′ and 5′-UAAAUACAUG CCCUGCUACTT-3′. The colorectal cancer cells were seeded in a 35-mm cell culture dish at density of 2 × 10^5^cells/well overnight. Before transfection, fresh medium containing 10% fetal bovine serum without antibiotics was replaced for 24 h. 200 pmol of Stealth™ CXCR4 siRNA or Control siRNA were diluted with 500 μL Opti-MEM® I reduced serum medium, then 10 μL of LipofectAMINE™ 2000 was diluted with 500 μl of Opti-MEM®I Reduced Serum Medium was kept at room temperature for 5 min, and then both of them were mixed quickly and stored at room temperature for another 20 min. Thereafter, the culture cells were directly added with the mixed solution of siRNA: Lipofectamine™ 2000 at a concentration of 100 nmol and mixed homogeneously, and then the mixture was placed and cultured in an incubator at 37 °C. The cells were harvested at 48 h after transfection for Western blot and subsequent experiments.

### Enzyme-linked immunosorbent assay

To evaluate IL-1α and IL-1Ra effcet of CXCL12 productions by fibroblast. The fibroblasts were seeded at a density of 2 × 10^5^ cells/ml into a 24-well plates allowed to adhere overnight, then, the medium was exchanged with 2% FBS. The culturing fibroblasts were stimulated by IL-1α (0–100 ng/ml) or 100 ng/ml of IL-1Ra, and fibroblasts were incubated for another 48 h. The medium were collected and microfuged at 1500 rpm for 5 min to remove the particles, and the supernatants frozen at − 80 °C until performed for enzyme-linked immunosorbent assay (ELISA). The concentration of CXCL12 was measured using an ELISA kit (R&D Systems) according to the instructions of manufacturer. To further investigate the synergistic effect of the tumor-stromal interaction, we examine the effect of colorectal cancer cell-derived IL-1α on CXCL12 production from fibroblasts using a double-chamber method in 24-well plates. The fibroblast was seeded at density of 2 × 10^5^cells/well into 24-well plates, and allowed to adhere overnight. The medium was exchanged with or without IL-1α or IL-1Ra, and co-cultured with 5 × 10^4^ cells/ml of HT-29 or CaCo-2 into inserts with 0.45-µm pores (Kurabo Co.). The co-culture systems were incubated for another 48 h, and subsequently CXCL12 concentration were measured as above described.

### In vitro proliferation of human umbilical vein endothelial cell in the presence of CXCL12 or anti-CXCL12 antibody

HUVEC was seeded at a density of 5 × 10^3^cells/100 µl in 96-well plates and allowed to adhere overnight, the medium were exchanged with medium alone (control) or media containing different concentrations of CXCL12 or 100 ng/ml of anti-CXCL12 antibody. After for 72 h incubation, HUVEC’s proliferation was determined by the WST-1 Cell Proliferation Assay System (Takara Bio Ing, Shiga, Japan). The absorbance was determined using a microplate reader (Molecular Devices, Sunnyvale, CA, USA) at a test wavelength of 450 nm and reference wavelength of 690 nm.

### The effect of proliferation of colorectal cancer cells on CXCL12 and CXCR4 siRNA

The colorectal cancer cells or transfected with CXCR4 siRNA cells were seeded at a density of 5 × 10^3^cells/100 µl in 24-well plates and allowed to adhere overnight, the medium were exchanged, the media containing different concentrations of CXCL12. After for 72 h incubation, the proliferation was determined by the WST-1 Cell Proliferation Assay System. The absorbance was determined using a microplate reader at a test wavelength of 450 nm and reference wavelength of 690 nm.

### In vitro invasion of colorectal cancer cells following pretreatment with CXCL12, CXCR4 siRNA or co-cultured with fibroblasts

The in vitro invasion assay was performed using BioCoat Matrigel Invasion Chambers (Bencton Dickinson, Bedford MA) according to the manufacturer’s instruction. First, the colorectal cancer cells transfected with or without CXCR4siRNA were seeded at density of 1 × 10^5^/ml cells into Martrigel pre-coated trans-wells containing of polycarbonate membranes with 8 µm pores. Tran-well chambers were then placed in 24-well plates with basic medium alone (control), or medium pretreated with 1, 10, 100 ng/ml of CXCL12. After 24 h incubation, the upper surfaces trans-wells were wiped by cottons and invaded cells were fixed and stained with Diff-Quik kit. The invaded cells were counted in five microscope fields (×100). To further investigate fibroblast-derived CXCL12 caused an increase on migration capability of colorectal cancer cells or HUVECs. The migration assay for HUVEC was performed using a double-chambers method. Fibroblast was seeded at density of 1 × 10^5^/ml cells into 24-well plates with FGM-2 medium, as same time, the trans-well chamber (containing colorectal cancer cells or HUVECs at 1 × 10^5^cells/chamber) were plated into 24-well plates allowed to incubate for 24 h, the invaded cancer cells or HUVECs were determined as above describe.

### Angiogenic activity of HUVEC pretreated with CXCL12

To investigate the influence of CXCL12 on tubular formation by HUVEC, HUVEC and fibroblast were co-cultured in basal medium only or in basal medium containing different concentration of CXCL12 using an Angiogenesis Kit (Kurabo Co.) according to the manufacturer’s protocols. Briefly, HUVECs and fibroblasts were co-cultured in 24-well plates with basal medium alone (control) or basal medium with 1 ng/mL, 10 ng/mL, 100 ng/mL of CXCL12 or 100 ng/mL anti-CXCL12 Ab. The culture medium was exchanged every two day, and cultured for a total of 11 days. HUVEC tubular formation was stained with anti-CD31 antibody according to the manufacturer’s protocols. The area of tubular formation was measured quantitatively over ten different fields for each condition using an image analyzer (Kurabo Co., Osaka, Japan).

### In vitro angiogenic activity during co-cultured with colorectal cancer cells

To further investigate the influence of colorectal cancer cell-derived IL-1α on tubular formation by HUVEC, the colorectal cancer cells (HT-29 secreted IL-1α or CaCo-2 not secreted IL-1α), HUVEC, and fibroblasts were co-cultured using a double-chamber method in 24-well plates. HT-29 or CaCo-2 cells (5 × 10^4^cells/ml) were seeded into trans-well chambers, consisting of polycarbonate membrane with 0.45-µm pores and allowed to adhere overnight. Trans-well chambers were then placed in the HUVEC/fibroblast co-culture system with or without 10 ng/mL of CXCl12, IL-1Ra or CXCR4 siRNA and exchanged on the sixth day. All cells were cultured for total 11 days. The HUVEC tubular formation was described as above. This assay allowed us to evaluate angiogenesis quantitatively and examine tumor-stromal interactions through soluble cytokines.

### Statistical analysis

Data are presented as means ± standard deviations (SD). Differences in the mean of two groups were analyzed by an unpaired *t* test. Multiple group comparison were performed by one-way ANOVA with a post hoc test for subsequent individual group comparisons. *P* < 0.05 was considered statistically significant. Mean values and SD were calculated for experiments performed in triplicate (or more).

## Results

### Expression of CXCL12, CXCR4 and IL-1α in colorectal cancer cells and fibroblasts

**Fig. 1:**
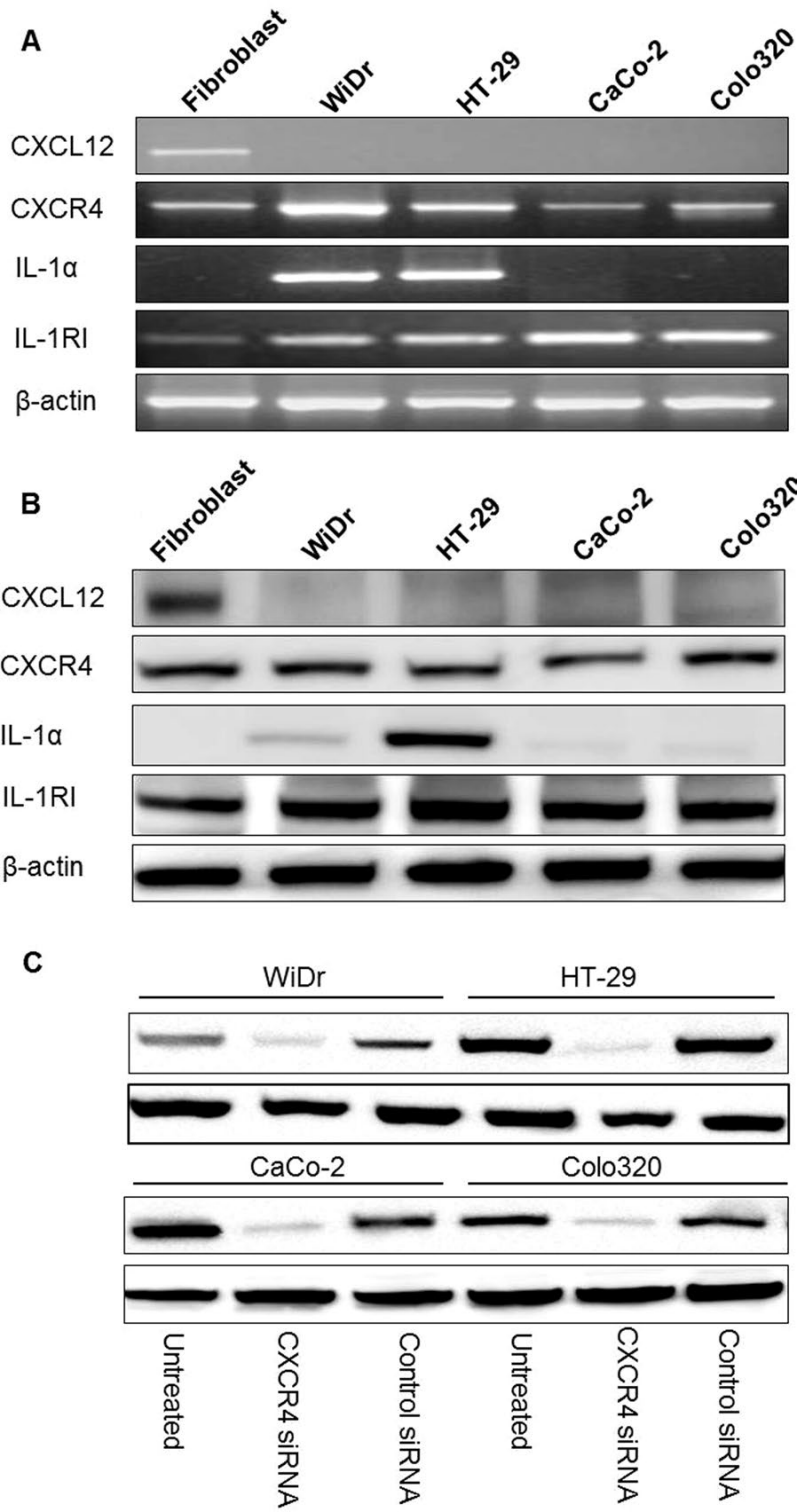
Expression levels of CXCL12, CXCR4, IL-1α and IL-1 RI in colorectal cancer cell lines and stromal cells, and siRNA blockage of CXCR4 expression in colorectal cancer cell lines. Expression levels of CXCL12, CXCR4 and IL-1α mRNA and protein in four human colorectal cancer cell lines and fibroblasts were detected by reverse transcription (RT)-PCR (**A**) and Western blotting (**B**), respectively. The CXCR4 protein expression level in four colorectal cancer cells transfected with CXCR4 gene-targeted small interfering RNA (CXCR4 siRNA) was detected by western blotting (**C**). β-actin was used as an control

We previously classified colorectal cancer cell lines into two groups; one is high liver metastatic cell lines (HT-29 and WiDr). The other is low liver metatstatic cell lines (CaCo-2 and CoLo320)^4^ by intrasplenic liver metastatic assay. RT-PCR and western blot revealed that IL-1α mRNA and protein were expressed by the higher liver-metastatic colorectal cancer cell lines HT-29 and WiDr, and not detected in low live-metastatic cell lines CaCo-2 and CoLo320. CXCL12 mRNA and protein were only expressed by fibroblasts. The CXCR4 and IL-1 RI mRNA and protein showed in all cell lines (Fig. 1A, B). The Silence of CXCR4 expression by siRNA pretreatment was confirmed by immunoblotting. Tranfection of CXCR4 siRNA led to a near total loss of CXCR4 expression of colorectal cancer cells. An anti-β-actin antibody served as control (Fig. 1C).

### Effect of recombinant human Il-1α and colorectal cancer cell-derived IL-1α on secretion levels of CXCL12 by fibroblasts

**Fig. 2:**
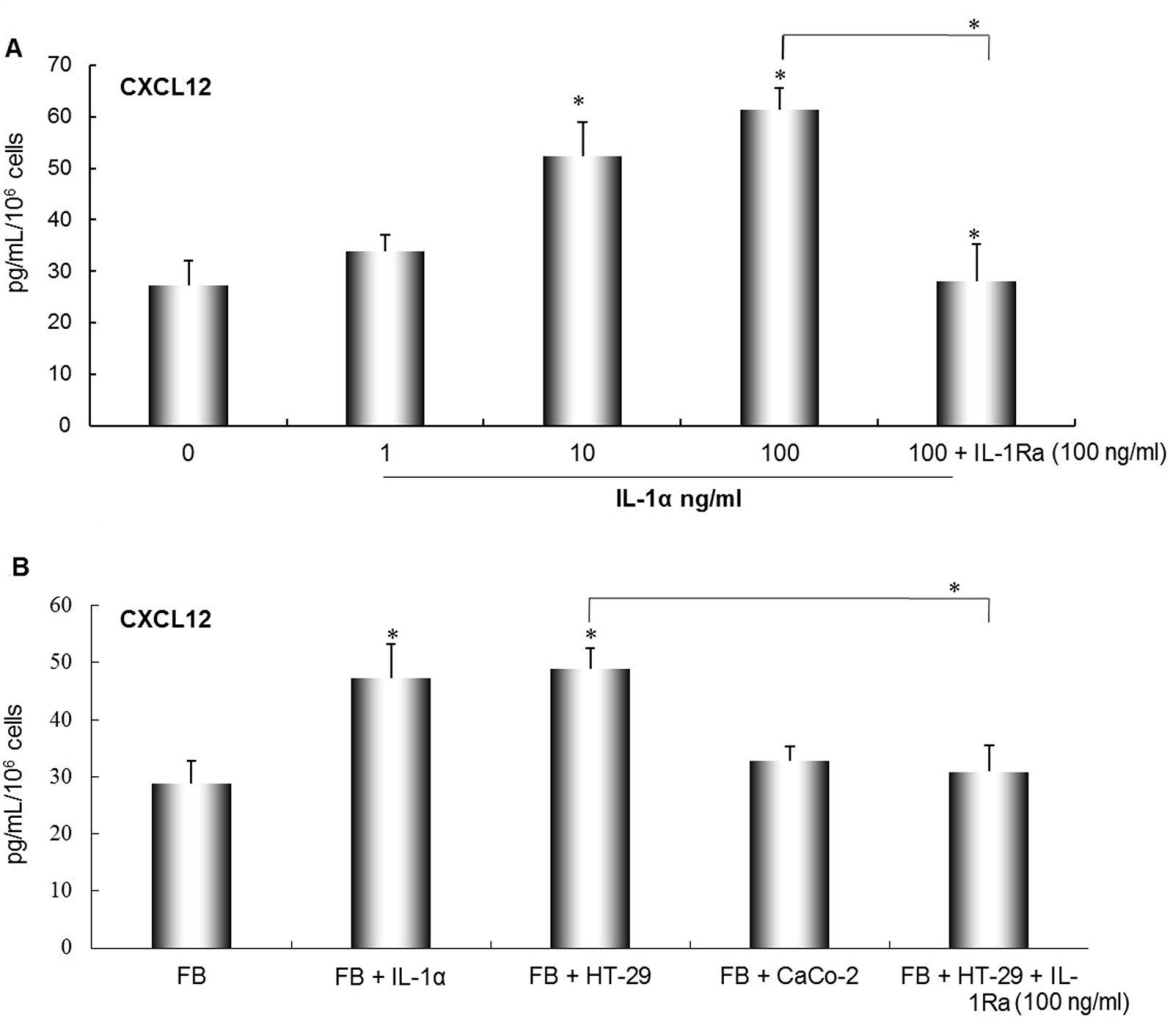
Recombinant human Il-1α (rIL-1α) and colorectal cancer cell-derived IL-1α effect on secreted CXCL12 levels by fibroblasts. CXCL12 protein concentration in cultured medium of fibroblasts was determined by ELISA. rIL-1α and IL-1Ra effect on secreted CXCL12 levels by fibroblasts (**A**). Co-cultured with colorectal cancer cells (HT-29 or CaCo-2) or IL-1Ra influence secreted levels of CXCL12 by fibroblasts (**B**). The values are expressed as mean ± SD. Multiple comparisons were performed by one-way ANOVA followed by student–Newman–Keuls test, *P < 0.01

The secreted CXCL12 of fibroblasts were increased by IL-1α in a dose-dependent manner, the promoted CXCL12 expression by IL-1α was significantly inhibited by the presence of IL-1receptor antagonist (IL-Ra) (*P < 0.01, Fig. 2A). Likewise, secretion level of CXCL12 by fibroblast were significantly enhanced by co-culturing HT-29, but have not significant effect of co-culturing with Caco-2, and treatment of IL-1α significantly increased secretion level of CXCL12 in this co-culture system. On the other hand, the enhanced CXCL12 production by co-culturing with HT-29 cells was significantly inhibited by the presence of IL-1Ra (*P < 0.01, Fig. 2B).

### Effect of CXCL12and anti-CXCL12 Ab on proliferation of colorectal cancer cells and vascular endothelial cell

**Fig. 3:**
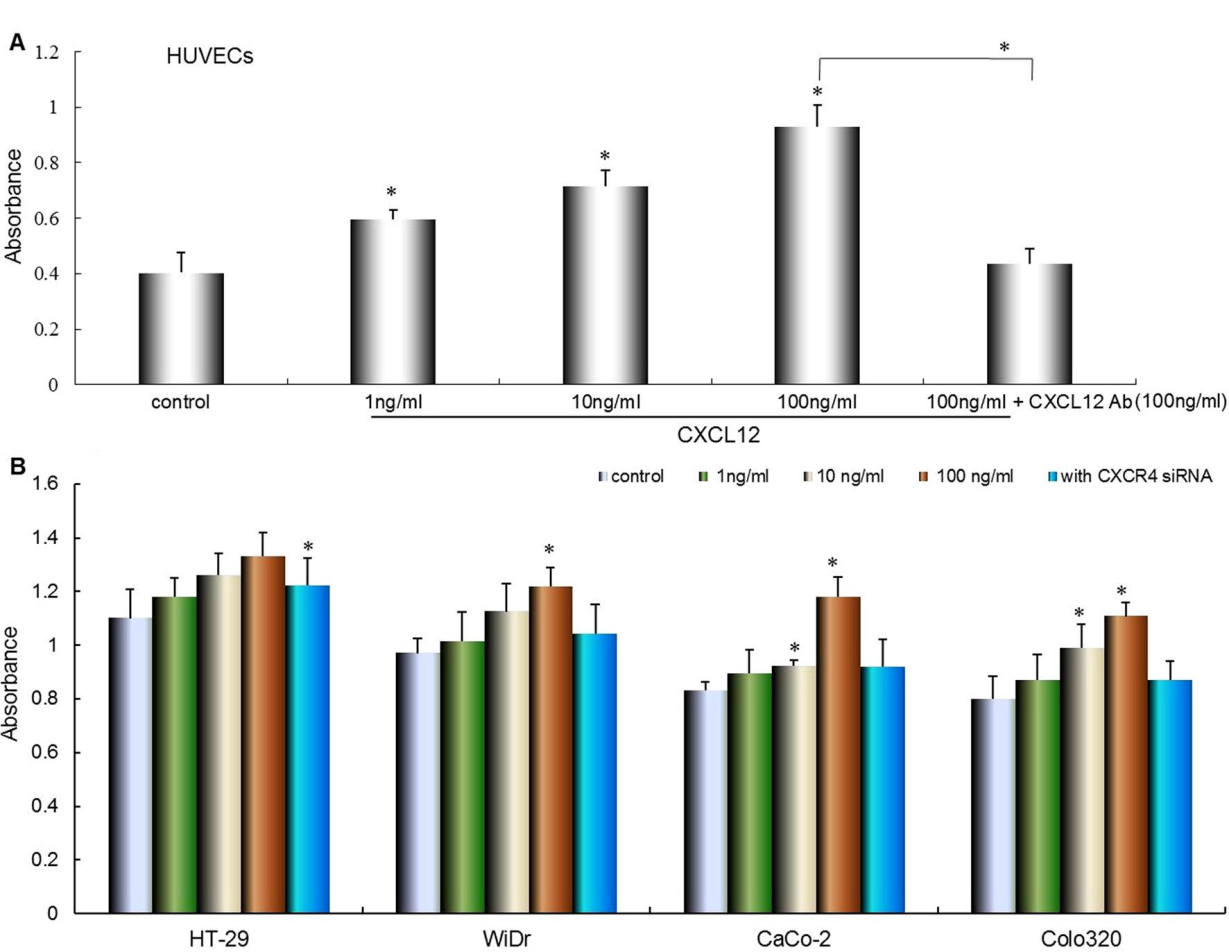
Effect of CXCL12 and CXCR4 gene silencing on the proliferation of HUVECs and colorectal cancer cells. **A** The effect of different concentration of recombinant CXCL12 on proliferation of HUVECs. HUVECs were cultured in medium containing different concentrations of CXCL12. After 72 h of incubation, the proliferation of colorectal cancer cells were assessed using premixed WST-1 cell proliferation assay (column mean absorbance reading; Bars:SD). Multiple comparisons were performed by one-way ANOVA followed by the Dunnett test. Bars indicate SD. *P < 0.01, compared with control (0 ng/ml). **B** The effect of different concentration of recombinant CXCL12 and CXCR4 siRNA on proliferation of colorectal cancer cells. HT-29, WiDr, CaCo-2 and Colo320 transfected with (or without) CXCR4 siRNA duplex oligoribonucleotide. The all cells were cultured in medium containing different concentrations of CXCL12. After 72 h of incubation, the proliferation of colorectal cancer cells were assessed by premixed WST-1 cell proliferation assay (column mean absorbance reading; Bars:SD). Multiple comparisons were performed by one-way ANOVA followed by the Dunnett test. Bars indicate SD. *P < 0.01, compared with control

To evaluate the effect of CXCL12 or Anti-CXCL12 Ab on proliferation of colorectal cancer cells and HUVECs, proliferation assay was performed by WST-1 cell proliferation assay. The result showed that proliferation of vascular endothelial cells (HUVECs) was also significantly enhanced by the addition of CXCL12 in a dose-dependent manner (*P < 0.01 compared with control), and this enhanced role was significantly blocked by anti-CXCL12 antibody (*P < 0.01 compared with control, Fig. 3A). The colorectal cancer cells proliferation were enhanced by CXCL12 in a concentration-dependent manner. The 100 ng/mL of CXCL12 was significantly promoted the proliferation of colorectal cancer cells. CXCR4 siRNA significantly inhibited the enhanced proliferation at presence of CXCL12 in culture media (*P < 0.01, Fig. 3B).

### Effect of CXCL12 or co-culture with fibroblast on colorectal cancer cell’s invasiveness

**Fig. 4:**
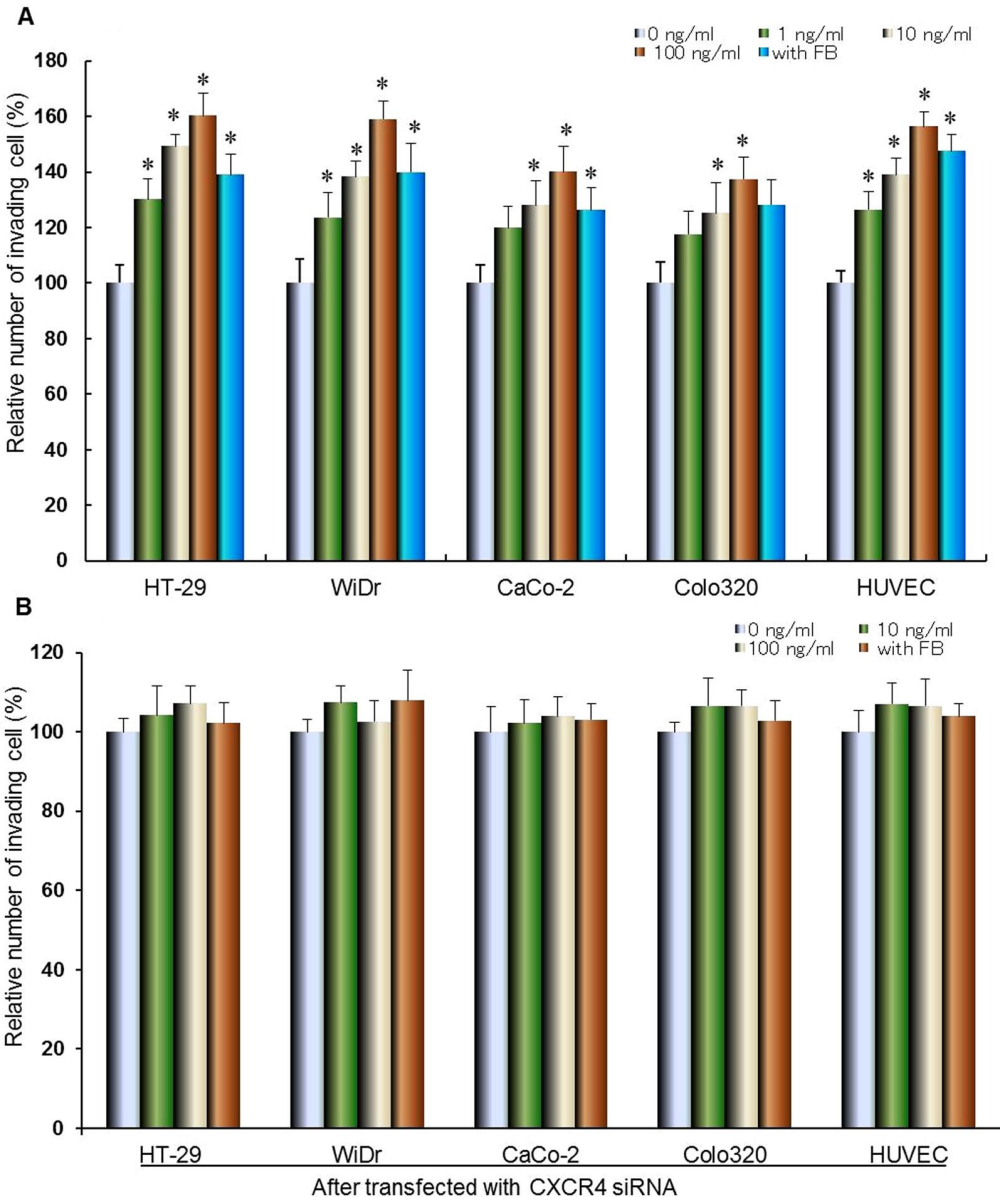
Effect of CXCL12 CXCR4 siRNA and co-cultured with fibroblasts on invasiveness of colorectal cancer cells. Colorectal cancer cells were treated with different concentration of CXCL12 or co-cultured with fibroblasts and incubated for 24 h, and then cell invasion were performed by Matrigel assay. Statistical significance was tested by one-way analysis of variance and post hoc test (Turkey Kramer multiple comparisons). All data are expressed as mean ± s.d, *P < 0.01compared with control (**A**). After transfected with CXCR4 siRNA or co-cultured with fibroblasts colorectal cancer cells were treated with different concentration of CXCL12 and incubated for 24 h, and then cell invasion were performed by Matrigel assay. Statistical significance was tested by one-way analysis of variance and post hoc test (Turkey Kramer multiple comparisons). Statistical significance was indicated by *P* < *0.05.* All data are expressed as mean ± s.d, *P < 0.01compared with control siRNA group (**B**)

To confirm the interaction between colorectal cancer and stromal cell-derived CXCL12 in tumor microenvironment, we next examined the effect of CXCL12 on colorectal cancer cell invasiveness using invasion assay. The invasive capability of colorectal cancer cells were enhanced by CXCL12 in a dose-dependent manner, 100 ng/mL of CXCL12 was significantly promoted cancer cells invasiveness (*P < 0.01). On the other hand, co-cultivation with fibroblasts caused significantly enhanced cancer cells invasion (*P < 0.01, Fig. 4A). After transfected with CXCR4 siRNA or co-cultured with fibroblasts, colorectal cancer cells were pretreated with different concentration of CXCL12 and incubated for 24 h, and then cell invasion were performed by Matrigel assay. The promotion of invasive capability of colorectal cancer cells by CXCL12 were blocked by CXCR4 siRNA (*P < 0.01compared with control, Fig. 4B).

### Effect of CXCL12 or co-culture with fibroblast on the migration of HUVEC

The migrating ability of HUVEC was enhanced by CXCL12 in a dose-dependent manner, and 100 ng/mL of CXCL12 was significantly enhanced HUVECs migration, but this enhanced role was inhibited by CXCL12 Ab. Co-cultivation with fibroblast caused significantly greater HUVEC migration (*P < 0.01) and this enhanced role was inhibited by CXCR4 siRNA (*P < 0.01, *P < 0.01compared with control, Fig. 4A, B).

### Effect of CXCL12 on the tube formation by HUVEC

**Fig. 5:**
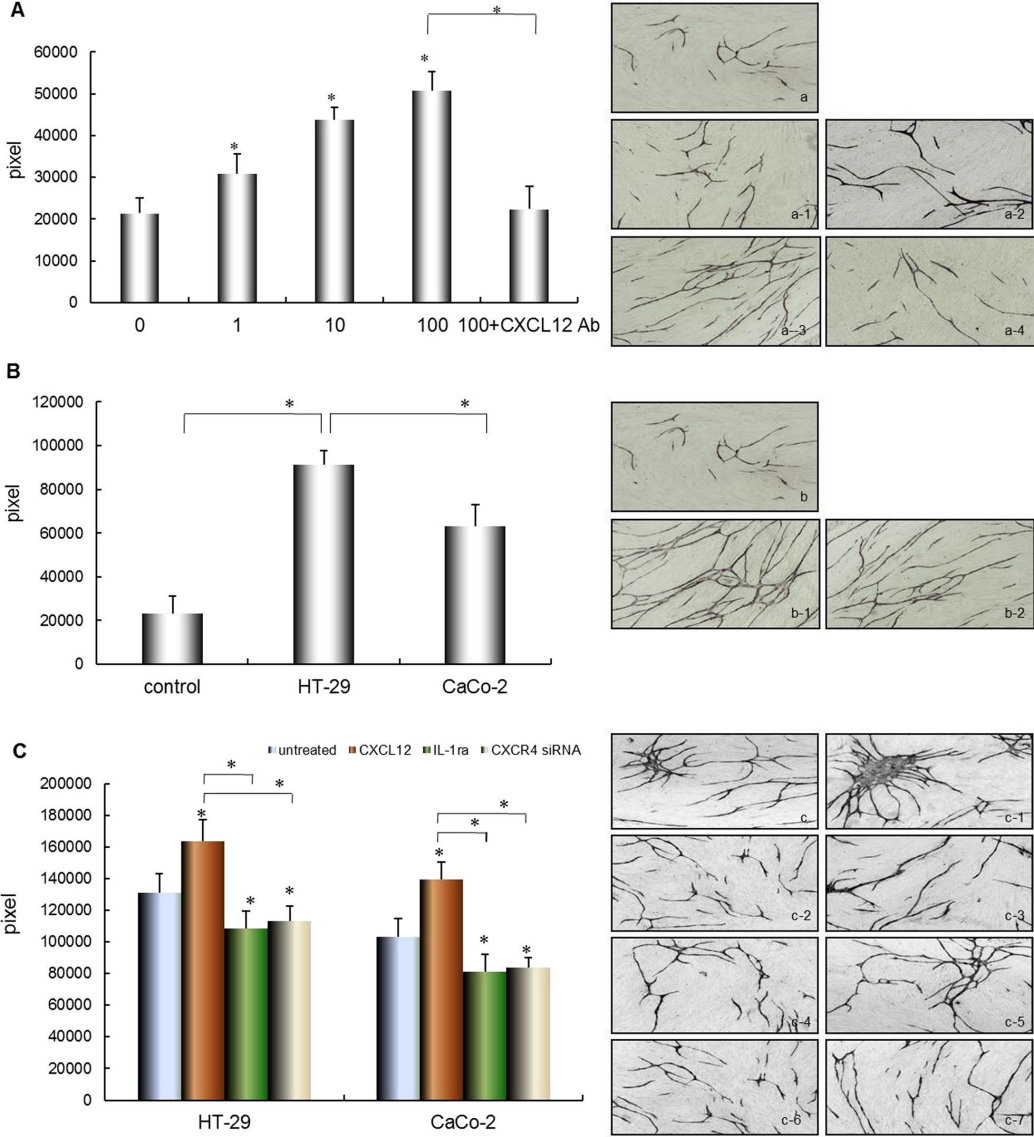
Effect of CXCL12, co-culture with colorectal cancer cells and IL-1Ra pretreatment on angiogenesis. (**A**) Effect of CXCL12 on angiogenesis. After incubation of HUVECs/fibroblasts in the presence or absence of CXCL12 or anti CXCL12 Ab for 11 d, angiogenesis were stained with CD31 antibody. (a: Control; a-1: Culture system treated with 1 ng/mL CXCL12; a-2: Culture system treated with 10 ng/mL CXCL12; a-3: Culture system treated with 100 ng/mL CXCL12; a-4: Culture system reated with 100 ng/mL CXCL12 and 1 μg/mL of anti CXCL12 Ab (Magnification: × 40). (**B**) Effect of co-culture with different metastatic potential colorectal cancer cells (HT-29) and (CaCo-2) on HUVEC tubular formation. HUVECs/fibroblasts were co-cultured with HT-29 or CaCo-2. Theco-cultured system was incubated for 11 days, and the tube formation was measured as described earlier. Magnification: × 200. (b: Control; b-1: co-cultured with HT-29 cells; b-2: c Co-cultured with CaCo-2 cells). (**C**) Effect of co-culture with HT-29 and CaCo-2 cells pretreated with CXCL12, IL-1Ra and CXCR4 siRNA on on HUVEC tubular formation. (c: co-cultured with HT-29 cells; c-1: co-cultured with HT-29 cells pretreated with 10 ng/mL CXCL12; c-2: co-cultured with HT-29 cells pretreated with 10 ng/mL of IL-1Ra; c-3: co-cultured with HT-29 cells tranfected with CXCR4 siRNA duplex oligoribonucleotide; c-4: co-cultured with CaCo-2 cells; c-5: co-cultured with CaCo-2 cells pretreated with 10 ng/mL CXCL12; c-6: co-cultured with CaCo-2 cells pretreated with 10 ng/mL of IL-1Ra; c-7: co-cultured with CaCo-2 cells tranfected with CXCR4 siRNA duplex oligoribonucleotide). Columns represent mean pixels of HUVEC tube formation area; error bars represent SD. Multiple comparisons were made by using one-way ANOVA, followed by Student–Newman–Keuls test. *P < 0.01

To ivestigate the role of CXCL12 in cell living microenvironment, we focused on the interaction between tumor cell and stromal cell. Thus, we characterized angiogenic activity in co-cultured fibroblasts and vascular endothelial cells and influenced by CXCL12. First, we examined the effect of CXCL12 on the formation of tube-like structures by HUVEC. The tubular formation was significantly enhanced by the presence of CXCL12 in a dose-dependent manner (*P < 0.01). Further, anti-CXCL12 inhibited tubular formation by HUVEC (*P < 0.01, Fig. 5A).

### Effect of colorectal cancer cells with or without IL-1α on tube formation by HUVEC

To further investigate the different metastatic potential colorectal cancer cells influence on tube formation by HUVEC. We cultured three cell lines using double chamber methods to determine the interaction among them. The tubular formation was significantly enhanced by co-culture with HT-29 cells compare to control (HUVECs and fibroblasts only) or co-culture with CaCo-2 cells, respectively (*P < 0.01, Fig. 5B). Moreover, the presence of IL-1Ra could significantly inhibit the tubular formation in co-culture with HT-29 or (*P < 0.01). The enhanced tubular formation by HUVEC was significantly inhibited by CXCR4 siRNA (*P < 0.01, Fig. 5C).

## Discussion

The liver metastasis of colorectal cancer is not only driven by the internal changes of tumor cells, but also closely related to the remodeling of tumor microenvironment. In the tumor microenvironment, chemokines can act on non-immune cells and vascular endothelial cells in the microenvironment, regulating the proliferation, invasiveness and metastasis of tumor cells [22]. CXC chemokine is mainly expressed in the stromal cells of tumor microenvironment and plays an important role in tumor progression, tumor-related inflammation immunity, and tumor invasion [23]. As one of the chemokines, CXCL12 and vascular endothelial growth factor (VEGF) have a synergistic effect on tumor angiogenesis [24]. At the same time, CXCL12 can also promote the proliferation and survival of tumor cells. In addition, the CXCL12-CXCR4 signal pathway composed of CXCL12 and its receptor CXCCR4 can promote the invasion and metastasis of many kinds of malignant tumors [25–27]. Our previous studies have shown that IL-1α is one of the most important inflammatory cytokines involved in the metastasis of colorectal cancer, and plays an important role in the metastasis of colorectal cancer. It promotes liver metastasis of colorectal cancer through IL-1α/PI3K/NF-κβ signal pathway. CXCL12 derived from stromal cells in the tumor microenvironment depends on PI3K/Akt/mTOR signal to up-regulate the secretion of CXCL6 or down-regulate the expression of PTEN through PI3k/Akt signal to enhance the liver metastasis of colorectal cancer. Is there a correlation between autocrine IL-1α and paracrine CXCL12 in colorectal cancer, and does this association affect liver metastasis of colorectal cancer? Can interleukin-1 receptor antagonist inhibit the occurrence of liver metastasis? Our study focuses on solving those problems.

Our results showed that IL-1α was expressed in high liver metastasis cell lines (HT-29 and WiDR) and human umbilical vein endothelial cells, CXCL12 was only expressed in fibroblasts, and CXCR4 was expressed in all cell lines. This suggests that IL-1α is associated with liver metastasis of colorectal cancer. It has also been proved that IL-1α is a pro-inflammatory and carcinogenic factor regulated by PGE2, which can stimulate the migration of colon cancer cells [28]. Moreover, cancer cell derived of IL-1α can promote the angiogenesis of pancreatic cancer cell lines with high metastasis to the liver, and actively regulate angiogenesis through the effect on interstitial cells of colon cancer, thus promoting the metastasis of distant organs, such as liver [29]. We further explored the relationship between IL-1α and the environment of colorectal cancer and its effect on metastasis, especially in angiogenesis. The results showed that exogenous and autocrine IL-1α could significantly increase the secretion of CXCL12 by the important stromal fibroblasts in tumor microenvironment, which could be inhibited by interleukin 1 receptor antagonist (IL-1Ra). CXCL12 can not only promote the proliferation of colorectal cancer cells, but also enhance the proliferation of vascular endothelial cells, which is positively correlated with the concentration of CXCL12. And CXCR4 siRNA regulates the proliferation of colorectal cancer and vascular endothelial cells by inhibiting the CXCR4/CXCL12 axis. In order to simulate the tumor microenvironment, we used a co-culture system composed of colorectal cancer cells or vascular endothelial cells and fibroblasts to detect the effects of stromal cell-derived CXCL12 on the invasion and migration of colorectal cancer and vascular endothelial cells. The results showed that CXCL12 derived from fibroblasts significantly enhanced the invasion of cancer cells and the migration of vascular endothelial cells, and this enhancement could be blocked by CXCL12Ab and CXCR4siRNA.

To investigate the role of CXCL12 in cancer cell living microenvironment, we focused on the interaction between tumor cell and stromal cell. Thus, we characterized angiogenic activity in co-cultured fibroblasts and vascular endothelial cells and influenced by CXCL12. First, we examined the effect of CXCL12 on angiogenesis by HUVEC. The angiogenesis was significantly enhanced by the presence of CXCL12 in a dose-dependent manner. Further, anti-CXCL12 inhibited tubular formation by HUVEC. To further investigate the different metatstatic potential colon cancer cells influence on tube formation by HUVEC. We cultured three cell lines using double chamber methods to determine the interaction among them. The tubular formation was significantly enhanced by co-culture with HT-29 cells compare to control (HUVECs and fibroblasts only) or co-culture with CaCo-2 cells, respectively. Moreover, the presence of IL-1Ra could significantly inhibit the tubular formation in co-culture with HT-29 cells system. Our results suggest that IL-1Ra inhibits liver metastasis of colorectal cancer by inhibiting CXCL12, secreted by fibroblasts in colorectal microenvironment and blocking CXCR4/CXCL12 signal to enhance the proliferation, invasion and neovascularization of colorectal cancer cells.

Similar findings have been reported for melanoma metastasis where a complete inhibition of lipopolysaccharide augmented hepatic metastasis by IL-1Ra was observed [30]. Meanwhile when treatment of mice with IL-1Ra can markedly inhibited the augmentation of lung metastasis to the human melanoma cell A375M in mice treated with endotoxin [31]. In addition, in clinical breast cancer model, blocking IL-1R with IL-1R antagonist (IL-Ra) can inhibit tumor progression, accompanied by decreased recruitment of myeloid cells. In the mouse model, IL-Ra could significantly reduce the percentage and total number of in tumor tissue [32]. These results suggest that IL-Ra reduces tumor growth and metastasis by regulating tumor microenvironment.

In conclusion, IL-1α and CXCL12 are not only important molecules in the interaction between colorectal cancer cells and tumor microenvironment, but also important cytokines affecting liver metastass of colorectal cancer. IL-Ra can inhibit the tumor-promoting effect of CXCR4/CXCL12 signal in the microenvironment of colorectal cancer by antagonizing the secretion of IL-1α in colorectal cancer cells, and then inhibit the liver metastasis of colorectal cancer. IL-1Ra is a potential target for the clinical treatment of liver metastasis of colorectal cancer. Using IL-1Ra alone or in combination with other targeted drugs may have better efficacy in the treatment of colorectal cancer, which will be our focus to explore in the future.

## Conclusion

Together, these results suggested that the autocrine IL-1α and paracrine CXCL12 co-enhances the metastatic potential of colorectal cancer cells; IL-1Ra can inhibit the metastatic potential of colorectal cancer cells via decrease IL-1α/CXCR4/CXCL12 signaling pathways.

## Abbreviations

CRC: Colorectal carcinoma
HUVEC: Human umbilical vein endothelial cell
IL-1α: Interleukin-1α
IL-1Ra: Interleukin-1 receptor antagonist
CXCL12: Chemokine (C-X-C motif) ligand 12
CXCR4: Chemokine (C-X-C motif) receptor 4
PI3K: Phosphatidylinositol 3-kinase
Akt: Protein kinase B
NF-κB: Nuclear factor κB
PGE2: Prostaglandin E2
PTEN: Phosphatase and tensin homolog deleted on chromosome ten
VEGF: Vascular endothelial growth factor
ERK1/2/: Extracellular signal-regulated kinase 1/2
Ets-1: E26 Transformation-specific sequence-1
